# Serum fetuin A level is associated with nonalcoholic fatty liver disease in Chinese population

**DOI:** 10.18632/oncotarget.22361

**Published:** 2017-11-10

**Authors:** Zhengsen Cui, Rong Xuan, Yunmei Yang

**Affiliations:** ^1^ Department of Geratology, The First Affiliated Hospital, College of Medicine, Zhejiang University, Hangzhou, 310003, China; ^2^ Department of Endocrine, The Third Hospital of Hangzhou, Hangzhou, 310000, China

**Keywords:** fetuin A, non-alcoholic fatty liver disease, metabolic disorder, insulin resistance, serum biomarker

## Abstract

**Objective:**

To investigate the association between serum fetuin A concentration and non-alcoholic fatty liver disease (NAFLD) in Chinese population.

**Methods:**

This case-control study enrolled 79 NAFLD cases and 79 non-NAFLD controls. All subjects were selected from Chinese population who received annual health examination in the First Affiliated Hospital of Zhejiang University in 2016. NAFLD was diagnosed mainly based on abdominal ultrasonography. The severity of NAFLD was categorized by serum level of alanine aminotransferase. Serum fetuin A was measured by ELISA.

**Results:**

Serum fetuin A level in NAFLD patients was significantly lower than that in controls (0.27±0.17 vs. 0.32±0.12g/L, *P* < 0.05). Compared with controls, mild NAFLD (0.24±0.16 g/L, P < 0.05) and moderate NAFLD (0.25±0.17 g/L, *P* < 0.05) had significantly lower concentration of Fetuin A, while Fetuin A level tended to slightly increase with the severity of NAFLD. The prevalence rate of NAFLD decreased (75 %, 40 %, and 36 %), as Fetuin A level elevated. ROC curve of Fetuin A was developed to predict the presence of NAFLD. Area under ROC was 0.656.

**Conclusion:**

Serum level of Fetuin A was lower in NAFLD patients than controls, while Fetuin A level increased with the severity of NAFLD, indicating a potential predicting role of Fetuin A in the development of NAFLD.

## INTRODUCTION

Non-alcoholic fatty liver disease (NAFLD) is a clinico-pathological syndrome with lipid accumulation and related pathological changes, in patients without excessive alcohol consumption. It encompasses a broad spectrum of liver injury, ranging from simple steatosis to nonalcoholic steatohepatitis (NASH), fibrosis, and cirrhosis [1]. The pathogenesis of NAFLD is not fully clear. The most widely supported theory is ‘two-hit’ hypothesis. Insulin resistance (IR) acting as the ‘first hit’, plays a key role in hepatic lipid accumulation. Other oxidative stressors give a ‘second hit’, initiating inflammatory cascades and fibrosis [2]. Currently, liver biopsy is the gold standard for NAFLD diagnosis, but not a perfect method due to its invasive nature and inevitable sampling bias. Non-invasive methods, including imaging studies and serologic tests, are emerging as new non-invasive methods in NAFLD diagnosis [1, 3].

Hepatokines are proteins exclusively or predominantly secreted by liver into circulation, which is known to contribute in glucose and lipid metabolism. Major hepatokines isolated now include Angiopoietin-related protein 6, Fetuin-A, Fibroblast growth factor-21 (FGF-21), Insulin-like growth factors (IGFs) and Insulin-like growth factor binding proteins (IGFBPs), Selenoprotein P, and Sex hormone-binding globulin, et al [4]. Their key effect in metabolism is modulating insulin sensitivity, which makes them able to serve as biomarkers of IR [5] and related metabolic disorders, such as NAFLD, type 2 diabetes mellitus (T2DM), and cardiovascular disease (CVD) [4].

Fetuin A (α2-HS-glycoprotein), one member of hepatokine family, is reported to be important regulator of metabolism [4]. In humans, Fetuin A gene is located on chromosomal 3q27, whose expression is found to be associated with metabolic syndrome and T2DM [6]. It has been reported that Fetuin A positively correlates with markers of early atherosclerosis [7], metabolic syndrome [8, 9] and IR [10, 11]. High circulating Fetuin A level was shown to be a strong predictor of incident T2DM [12, 13] and cardiovascular events as well [14], independent of other well-established risk factors, which indicate its role in the pathophysiology of T2DM and CVD [4]. However, in some studies it was found that circulating Fetuin A levels were elevated in humans with hepatic fat accumulation [15-17], but there has been different findings [18].

Herein, we performed a case-control study to investigate the association between serum fetuin A level and NAFLD in Chinese population.

## RESULTS

**Figure 1:**
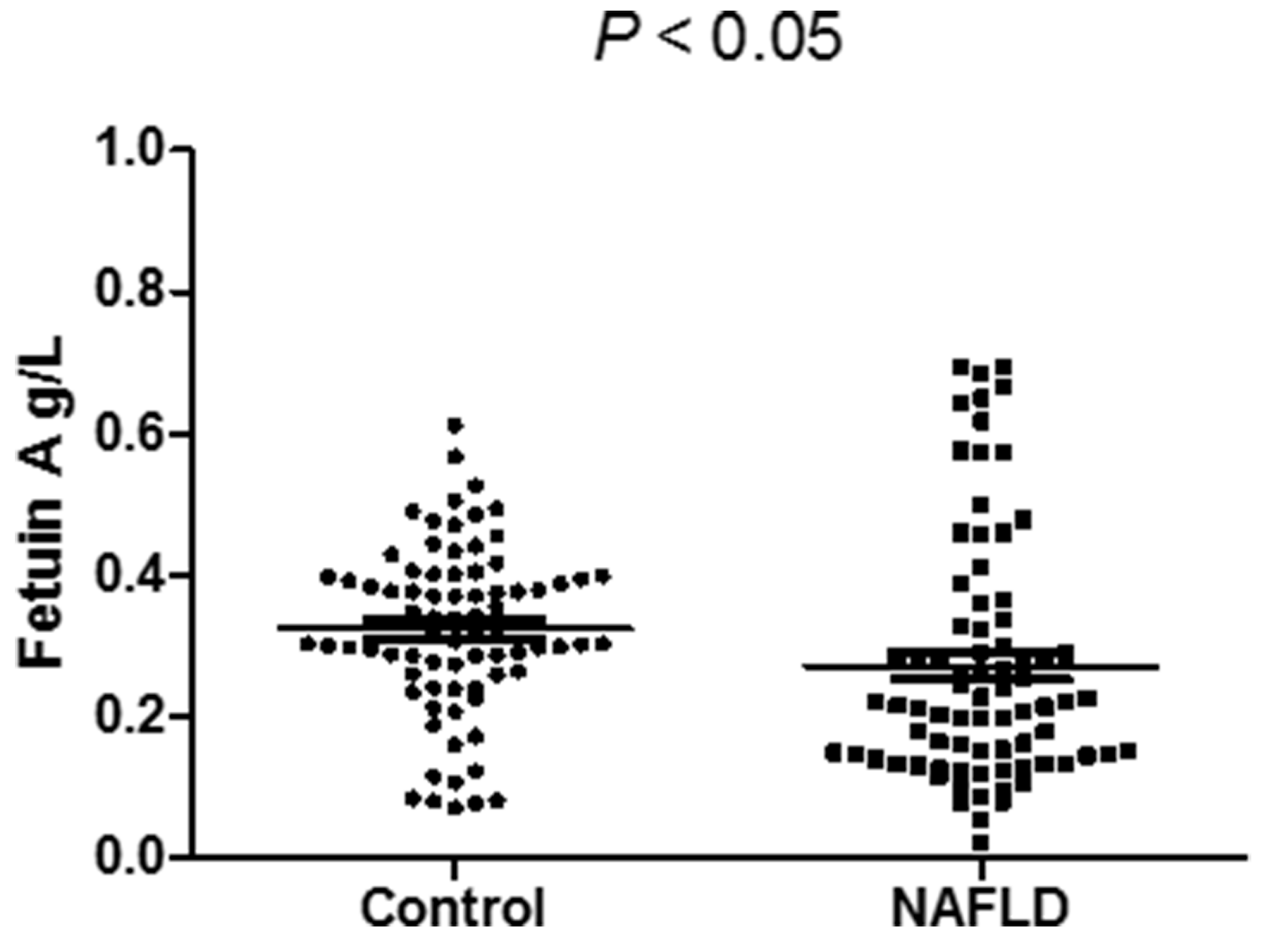
Serum fetuin A level is significantly lower in NAFLD

A total of 158 subjects were included in this study. The baseline characteristics are presented in Table 1. Serum level of Fetuin A in NAFLD patients (0.27±0.17 g/L, *P* < 0.05) was significantly lower than that in controls (0.32±0.12 g/L, Table 1 ; Figure 1). The mean age of subjects was 41.4 years and 73.4% of them were males. As expected, NAFLD patients presented with a higher BMI than age and gender-matched controls, as well as SBP and DBP (Table 1). In addition, subjects with NAFLD had higher serum levels of liver enzymes (ALT, AST, ALP and GGT), TG, TC, LDL-C, FPG, uric acid, and WBC, while lower levels of HDL-C compared to controls (Table 1). HOMA-IR was significantly higher in NAFLD patients (*P* < 0.001, Table 1).

**Table 1:** Baseline characteristics

Variables	Control	NAFLD	*P*_*trends*_
n (male/female)	79(58/21)	79(58/21)	1.000
Age (year)	40.0±12.0	42.8±10.8	0.094
Body mass index (kg/m^2^)	22.0±2.0	26.0±3.0	< 0.001
Systolic blood pressure (mmHg)	117±15	128±13	< 0.001
Diastolic blood pressure (mmHg)	71±9	79±10	< 0.001
Alanine aminotransferase (U/L)	21±13	32±21	< 0.001
Aspartate aminotransferase (U/L)	21±6	24±8	< 0.01
Alkaline phosphatase (U/L)	68±19	76±19	< 0.05
γ-Glutamyltransferase (U/L)	24±18	45±43	< 0.01
Total bilirubin (μmol/L)	12.7±5.3	13.5±5.8	0.337
Triglyceride (mmol/L)	1.17±0.63	2.12±1.40	< 0.001
Albumin (g/L)	48.7±3.0	49.2±3.3	0.327
Total cholesterol (mmol/L)	4.49±0.91	5.15±0.86	< 0.01
HDL cholesterol (mmol/L)	1.39±0.37	1.22±0.26	< 0.05
LDL cholesterol (mmol/L)	2.46±0.74	2.96±0.67	< 0.05
Fasting plasma glucose (mmol/L)	4.76±0.51	5.01±0.85	< 0.05
Serum uric acid (μmol/L)	344±77	380±87	< 0.01
White blood cell (×10^9^/L)	6.4±1.7	7.2±1.6	< 0.01
Platelet count (×10^9^/L)	228±53	236±46	0.291
HOMA-IR	1.81±1.80	3.27±2.18	< 0.001
Serum fetuin A (g/L)	0.32±0.12	0.27±0.17	< 0.05

^*^mean ± SD

**Figure 2:**
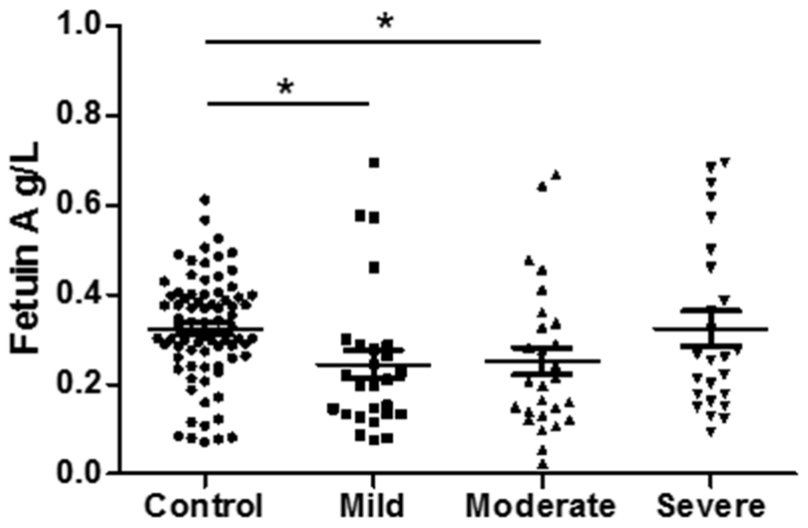
Serum fetuin A level is associated in NAFLD severity

All 158 subjects were divided into four groups (controls, mild, moderate and severe NAFLD), according to of serum ALT level, as indicated in Table 2. Figure 2 shows the comparisons of serum fetuin A levels among the four groups. Compared with controls (0.32±0.12 g/L), mild NAFLD (0.24±0.16 g/L, *P* < 0.05) and moderate NAFLD (0.25±0.17 g/L, *P* < 0.05) had significantly lower levels of Fetuin A, while serum fetuin A level tended to increase with the severity of NAFLD.

**Table 2:** Serum fetuin A level is associated with NAFLD severity

Variables	Control	Mild	Moderate	Severe	*P*_*trends*_
Alanine aminotransferase (U/L)	21±13	16±3	26±4	54±22	< 0.001
Serum fetuin A (g/L)	0.32±0.12	0.24±0.16	0.25±0.17	0.32±0.19	< 0.05
No. of subjests	79	26	26	27	
Age (year)	40.0±12.0	42±12	47±10	39±10	< 0.05
n (male/female)	79(58/21)	15/11	20/6	23/4	0.148
Body mass index (kg/m^2^)	22.0±2.0	23.3±1.63	25.0±3.1	27.9±2.8	< 0.001
Systolic blood pressure (mmHg)	117±15	124±18	128±10	132±7	< 0.001
Diastolic blood pressure (mmHg)	71±9	75±12	79±10	84±6	< 0.01
Aspartate aminotransferase (U/L)	21±6	25±6	27±11	21±7	< 0.001
Alkaline phosphatase (U/L)	68±19	71±18	82±14	74±23	< 0.05
γ-Glutamyltransferase (U/L)	24±18	46±35	54±42	34±49	< 0.001
Total bilirubin (μmol/L)	12.7±5.3	15.0±6.6	13.7±5.2	12.0±5.3	0.182
Triglyceride (mmol/L)	1.17±0.63	2.40±1.07	2.62±1.82	1.65±1.10	< 0.001
Albumin (g/L)	48.7±3.0	49.3±3.0	48.4±2.7	49.7±4.1	0.356
Total cholesterol (mmol/L)	4.49±0.91	5.24±1.09	5.43±0.66	4.93±0.76	< 0.01
HDL cholesterol (mmol/L)	1.39±0.37	1.09±0.20	1.22±0.29	1.31±0.25	< 0.05
LDL cholesterol (mmol/L)	2.46±0.74	3.13±0.81	3.10±0.57	2.78±0.61	< 0.01
Fasting plasma glucose (mmol/L)	4.76±0.51	4.85±0.43	5.27±1.24	4.92±0.61	< 0.05
Serum uric acid (μmol/L)	344±77	402±79	398±82	342±88	< 0.01
White blood cell (×10^9^/L)	6.4±1.7	7.2±1.6	7.5±1.7	7.0±1.5	< 0.05
Platelet count (×10^9^/L)	228±53	246±51	228±46	236±41	0.397
HOMA-IR	1.81±1.80	2.80±2.05	3.46±2.24	3.56±2.24	< 0.001

**Figure 3:**
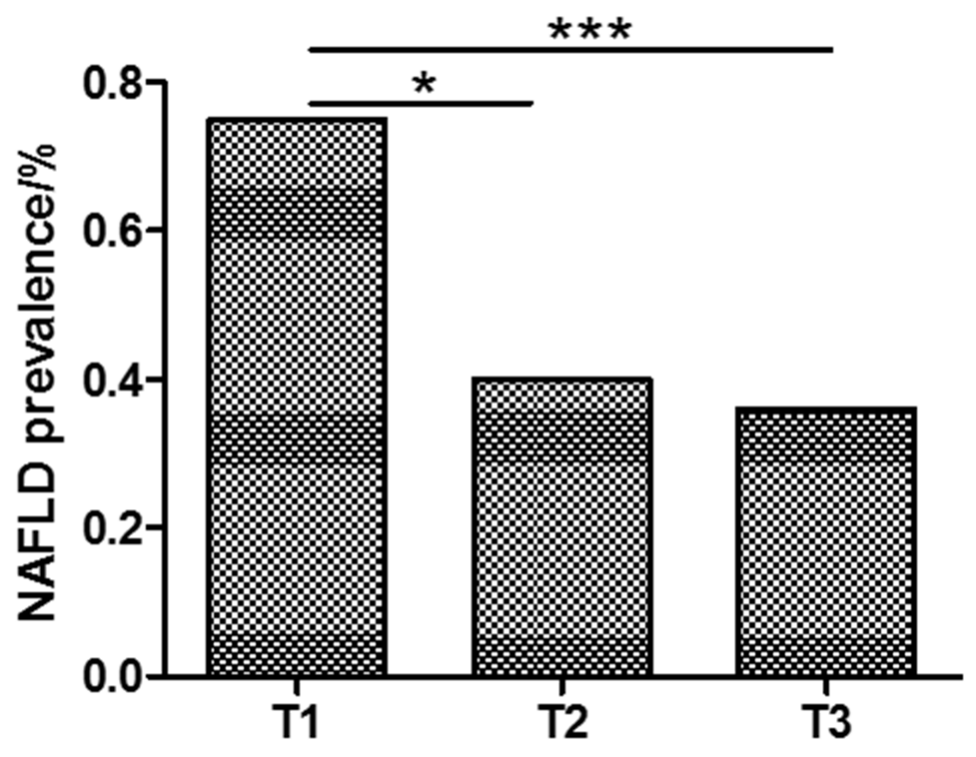
NAFLD is more prevalent in lower Fetuin A group

Based on the textiles of serum fetuin A level, all 158 subjects were divided into three groups (T1, T2, and T3), as shown in Table 3. NAFLD proportion presented a significant difference among the three groups (*P* < 0.001) that the prevalence rate of NAFLD decreased (75 %, 40 %, and 36 %), as Fetuin A level elevated (Figure 3). In addition, BMI (*P* < 0.05) decreased and HOMA-IR showed a decrease-increase trend (*P* < 0.01) as Fetuin A level elevated (Table 3).

**Table 3:** NAFLD proportion is associated with Fetuin A tertiles

Variables	T1	T2	T3	*P*_*trends*_
Serum fetuin A (g/L)	0.14±0.05	0.28±0.03	0.47±0.10	< 0.001
No. of subjests	52	53	53	
NAFLD n (%)	39(75)	21(40)	19(36)	< 0.001
Age (year)	41.5±1.6	41.4±12.2	40.9±10.6	0.963
Male, n (%)	35(67)	45(85)	36(68)	0.068
Body mass index (kg/m^2^)	24.6±1.6	24.3±3.6	22.4±3.2	< 0.05
Systolic blood pressure (mmHg)	126±9	122±16	120±15	0.285
Diastolic blood pressure (mmHg)	76±9	75±13	74±10	0.795
Alanine aminotransferase (U/L)	25±12	27±18	27±22	0.864
Aspartate aminotransferase (U/L)	24±9	22±6	22±7	0.220
Alkaline phosphatase (U/L)	75±19	72±21	70±17	0.417
γ-Glutamyltransferase (U/L)	40±44	31±26	33±30	0.368
Total bilirubin (μmol/L)	13.1±4.9	12.3±6.2	13.9±5.4	0.330
Triglyceride (mmol/L)	1.85±1.38	1.79±1.12	1.30±0.84	0.135
Albumin (g/L)	48.7±3.0	49.1±2.8	48.9±3.7	0.791
Total cholesterol (mmol/L)	4.91±1.05	4.83±0.85	4.73±0.92	0.765
HDL cholesterol (mmol/L)	1.23±0.24	1.27±0.41	1.42±0.29	0.055
LDL cholesterol (mmol/L)	2.87±0.83	2.64±0.73	2.62±0.66	0.335
Fasting plasma glucose (mmol/L)	4.95±0.96	4.86±0.65	4.84±0.43	0.717
Serum uric acid (μmol/L)	360±73	359±93	368±86	0.845
White blood cell (×10^9^/L)	7.2±1.5	6.6±1.8	6.7±1.8	0.152
Platelet count (×10^9^/L)	237±43	227±47	233±59	0.543
HOMA-IR	3.25±2.17	1.94±2.14	2.46±1.89	< 0.01

**Figure 4:**
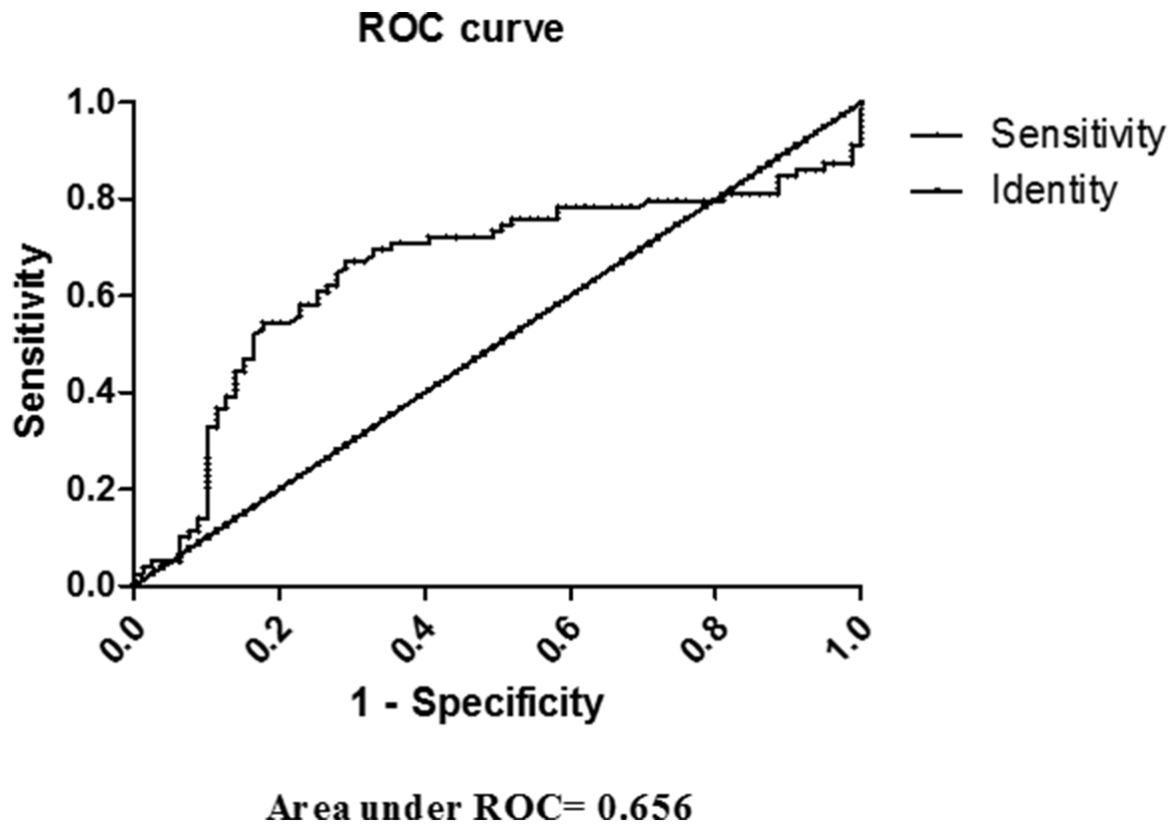
ROC curve of Fetuin A in diagnosing NAFLD

ROC curve of Fetuin A is developed to determine the best cut-off point to improve its diagnostic value of NAFLD (Figure 4). Area under ROC was 0.656, indicating a predictive role of Fetuin A in NAFLD.

## DISCUSSION

In this study, NAFLD patients was shown to have significantly lower serum level of Fetuin A than controls in Chinese population, while Fetuin A level tended to increase with the severity of NAFLD. Additionally, the prevalence rate of NAFLD increased, as Fetuin A level elevated among its textiles.

First, our study population showed typical serological changes as previously reported, represented by higher BMI, SBP and DBP, liver enzymes (ALT, AST, ALP and GGT), liver lipids (TG, TC and LDL-C), FPG, uric acid, and HOMA-IR [19-21]. Previous studies have revealed a dual role of Fetuin A in NAFLD. Several article reported that circulating Fetuin A level was elevated in NAFLD, whether for adults or children [17, 19, 20]. However, in Japanese population, serum fetuin A level is found to be negatively associated with liver/vessel fibrosis-related markers in NAFLD patients, indicating that circulating Fetuin A could be a useful serum biomarker for predicting liver and vascular fibrosis progression in NAFLD patients [18]. In previous basic scientific research, Fetuin A was demonstrated to induce IR and activated inflammatory response in NAFLD, indicating an adverse role in NAFLD [22].

Hypothesis has been raised referred to decease-increase trend in serum fetuin A level as NAFLD exacerbated. First, Fetuin A was indeed an aggravated element in NAFLD, and down-regulation might be explained by feed-back protection mechanism when steatosis first appeared and cell injury confined to mild range. Second, as disease severity increased, compensatory pathways weakened, and Fetuin A as well as other elevated. Third, our study subjects was included from annual health examination groups, relatively mild NAFLD, characterized by simple steatosis was identified among all NAFLD patients with no steatohepatitis or fibrosis. So, in general, our population was symbolled by decreased serum fetuin A level. Further populational and basic scientific research are in great need to interpret causal relationship and mechanism in this concern.

There are some limitations in our study. Firstly, all subjects are of health examination population from a single center. In the future, a wider population from multiple centers is needed. Secondly, the diagnosis of NAFLD is based on ultrasonography and the severity of NAFLD is divided by serum ALT level, which were non-invasive, convenient and widely used in clinical studies, despite of not being gold standard as live biopsy.

Our study provides a new way for diagnosis and therapeutic target of NAFLD. Fetuin A, as an important regulator of lipid metabolism, contributes to the development of NAFLD. As serum fetuin A level is lower in mild cases and changes with the severity of NAFLD, it can be used in early diagnosis and disease severity assessment combined with other methods (e.g. ultrasonography). Moreover, besides weight loss, medication regulating the serum level of Fetuin A antagonist might be effective for NAFLD treatment. Haukeland JW et al. found that plasma Fetuin A level decreased significantly after metformin treatment compared with placebo [17]. Jung TW et al. revealed that salsalate and adiponectin ameliorated hepatic steatosis via Fetuin A inhibition through AMPK-NFκB pathway [23]. Based on this, further clinical trials for drugs are required, as well as new therapeutic research.

In conclusion, this case-control study in Chinese population demonstrated that serum level of Fetuin A was lower in NAFLD patients than it in controls, while Fetuin A level increased with the severity of NAFLD. It suggested Fetuin A as a potential biomarker in the development of NAFLD.

## MATERIALS AND METHODS

### Study design and subjects

79 NAFLD cases and 79 non-NAFLD controls were included in this case-control study. The subjects were adults who received annual health examination in the First Affiliated Hospital of Zhejiang University in 2016. Subjects were excluded if they had excessively alcoholic consumption (men > 140 g/week or women > 70 g/week), viral/drug-induced/autoimmune liver diseases, malignant tumor, severe cardiopulmonary disorders, renal dysfunction, severe inflammatory diseases, thyroid dysfunction and pregnancy. This study was approved by the Ethics Committee of the First Affiliated Hospital of Zhejiang University in accordance with the Helsinki Declaration. All subjects gave informed consent before enrollment.

### Diagnostic criteria of NAFLD

NAFLD was diagnosed according to the Guidelines for the diagnosis and management of NAFLD recommended by the Chinese Liver Disease Association [24]. As an important diagnostic method, abdominal ultrasonography was performed for every subject by experienced sonologists using a Toshiba Nemio 20 sonography machine with a 3.5-MHz probe (Toshiba, Tokyo, Japan). In addition, the severity of NAFLD was classified into three grades (mild, moderate, and severe) according to the serum level of alanine aminotransferase.

### Anthropometric and serologic examinations

Anthropometric and serologic examinations were performed as previously described [21]. Demographic data, including gender, age, height, body weight, medical history and family medication were recorded. Body mass index (BMI) was calculated as body weight (in kilograms) divided by square of height (in meters). Systolic blood pressure (SBP) and diastolic blood pressure (DBP) were measured with a sphygmomanometer in sitting position. Meanwhile, overnight fasting blood samples were collected from each subject, and serologic tests were performed using a Hitachi 7600 autoanalyzer (Hitachi, Tokyo, Japan). Serum levels of alanine aminotransferase (ALT), aspartate aminotransferase (AST), alkaline phosphatase (ALP), γ-glutamyltransferase (GGT), total bilirubin (TB), triglycerides (TG), albumin, total cholesterol (TC), HDL cholesterol (HDL-C), LDL cholesterol (LDL-C), fasting plasma glucose (FPG), serum uric acid (UA) were recorded, as well as white blood cell (WBC) and platelet count. IR index (homeostasis model assessment for IR, HOMA-IR) was calculated as [fasting insulin (mIU/mL) × fasting plasma glucose (mmol/L)]/22.5 [25]. Serum fetuin A level was measured by a commercial ELISA (No. SEA178Hu; Cloud-Clone Corp., Houston, TX, USA).

### Statistics analysis

All statistical analyses were performed using SPSS 20.0 (SPSS Inc., Chicago, IL, USA). Normality of distribution was tested with Kolmogorov–Smirnov test. Normally distributed variables were presented as mean ± standard deviation (SD); variables with skewed distributions were presented as median (interquartile range). Student’s *t*-test or Mann–Whitney *U* test for continuous variables, and χ^2^ test for categorical variables were used to compare the parameters between cases and controls. For comparisons among various groups, one-way ANOVA or Kruskal-Wallis test was performed. Receiver operating characteristic (ROC) curve of Fetuin A was developed to help diagnosing NAFLD. A two-sided *P* < 0.05 was considered statistically significant.

## Abbreviations

BMI: Body Mass Index
WC: Waist circumference
FBG: Fasting blood glucose
FINS: Fasting insulin
HOMA-IR: Homeostasis model insulin resistance index
TG: Triglycerides
TC: Total cholesterol
HDL-C: High density lipoprotein cholesterol
LDL-C: Low density lipoprotein cholesterol
UA: Uric acid
